# Influencing Factors on College Students' Willingness to Spread Internet Public Opinion: Analysis Based on COVID-19 Data in China

**DOI:** 10.3389/fpubh.2022.772833

**Published:** 2022-02-18

**Authors:** Pinghao Ye, Liqiong Liu, Joseph Tan

**Affiliations:** ^1^School of Information Engineering, Wuhan Business University, Wuhan, China; ^2^DeGroote School of Business, McMaster University, Hamilton, ON, Canada

**Keywords:** adult attachment (AA), COVID-19, emotional motivation (EM), epidemic prevention, public opinion, social motivation (SM)

## Abstract

Following COVID-19 outbreak, Internet public opinion has tended to proliferate. From a theoretical perspective, however, the spread law of Internet public opinion in major epidemic prevention and control may provide optimization strategies on how best to channel Internet public opinion. Specifically, this article aims at exploring key factors affecting our theoretical understanding on the spread of Internet public opinion on a major epidemic situation amongst college students. A questionnaire survey on college students was conducted *via* online research data collection platform located in Changsha, China, amassing three hundred and nineteen valid questionnaires. Smart PLS was applied to verify a theoretical model vis-à-vis the reliability and validity of the measuring instrument. Results show that adult attachment and social motivation have significant positive influences on the consciousness of social participation. Evidently, adult attachment, emotional orientation and risk perception also have significant positive influences on emotional motivation. Emotional motivation plays a mediating role in the relationship between affective disposition and dissemination willingness. Additionally, social motivation, consciousness of social participation and emotional motivation significantly influence one's dissemination willingness in a positive way. The consciousness of social participation plays a mediating role in the relationship between social motivation and dissemination willingness. Social motivation plays a moderating role in the relationship between risk perception and dissemination willingness. Altogether, theoretical rationalization to enhance understanding and guide the initiation and spread of Internet public opinion of major public health emergencies accurately has now been provided by this work.

## Introduction

Today, the Internet comfortably eases users in gathering a wealth of information and has oftentimes become the main choice and the vehicle that people use to seek health knowledge (1). Even so, there are risks and hidden dangers; for instance, online rumors and fake health news have flooded public opinion forums. Various published articles on health, spread of epidemics and treatment methods have appeared on social media; sadly, the sources and contents of these articles are often vague and may not be easily affirmed.

Distinguishing facts (the truth) from rumors (the fake) has become challenging. Information receivers often have difficulties finding the truth (2, 3). When health-related rumors wantonly spread and behave like viruses, they will instill unnecessary anxiety to users and instigate troubles onto their lives (4). Inaccurate health information from some alarmists will arouse the public's psychological panic and gradually destroy the social trust system. Accordingly, researchers should attach great importance to studying the spread of health information.

COVID-19 is a notable type of acute respiratory tract infectious disease with entire populations to be generally susceptible. On January 30th, 2020, the World Health Organization (WHO) declared that COVID-19 was an international public health emergency (5). COVID-19 poses a great threat to the safety as well as physical and mental health of the public due to its high infectiousness, deadliness and uncertain treatment strategies (6). During a period of major epidemic prevention and control, the Internet has become a key channel for the masses to understand the progress of epidemic prevention and control and information related to the epidemic situation (7). Without a rational, scientifically sound, and effective public opinion guidance and governance, public opinion expressed on the Internet may evolve into informed public opinion, and the crisis caused by it is equivalent to the threat and panic from major epidemic situation. Accordingly, the internet public opinion on a major epidemic and its governance practice should be studied meticulously and particularly from a theoretical perspective as purposed by the effort presented herein.

The effect of COVID-19 on the social economy and psychology of the masses has attracted the attention of many scholars, including the influence of COVID-19 on citizens' psychology (8); the effect of COVID-19 on the public health management (9); and the effect of COVID-19 on the regional and national economic development (10). Since the outbreak of COVID-19, major Internet events have endlessly emerged, and Internet public opinion has been rapidly formed and fermented. College students, as a special group, are the hope of each family and respective country. These students are the frontier group clearly exposed to the use of new technologies and opened to new ideas in society owing to their active thinking and skilful use of the Internet for gathering new information. College students in contact with new things can often quickly accept information and adapt to the changes in the environment.

The psychosocial cognition of these students on COVID-19 is relatively high (11). To a great extent, the cognition and attitude of college students toward the epidemic situation not only determine their own physical and mental health but also affect the surrounding population (11). Focusing on college students' knowledge, attitude and behavior on the COVID-19 epidemic situation and understanding the coping style of college students in major epidemic prevention and control from a theoretical perspective can therefore provide key information anchors for understanding major epidemic prevention and control. These mechanisms are also conducive to endorsing the significant role of college students in major epidemic prevention and control. The public can correctly understand the knowledge on major epidemic situation and improve the self-protection ability *via* extensive and in-depth health education. Accordingly, the normal social order may be maintained. Such approach can also promote the protection of public health emergencies (12).

In this work, we take college students as the objects to be investigated. We analyse the spread characteristics of Internet public opinion (IPO) in the prevention and control of COVID-19 and construct a structural equation model vis-à-vis a constructive theoretical approach. Moreover, we deploy an empirical analysis to validate those key theoretical factors affecting the IPO spread on a major epidemic situation amongst college students. Hence, this work offers a much-needed theoretical support for the government (and other policymakers) in the management of IPO and the formulation of countermeasures.

This study comprises eight parts. First, the research background, research significance and research content of key influencing factors on IPO on a major epidemic prevention and control are introduced. Second, the research literature on IPO regarding major epidemic prevention and control is summarized. Here, a theory-based research model of influencing factors affecting college students' IPO spreading on major epidemic prevention and control is constructed. Third, research hypotheses are proposed and rationalized. Fourth, the variables and measurement questions embedded in the research model are highlighted. As well, we determine the questionnaire and research method, and explain the data analytical method to be applied in the study.

Fifth, the valid questionnaire samples collected are analyzed and the conclusion of this paper is drawn. Sixth, we systematically analyse and summarize the conclusion of this study. Seventh, the major theoretical significance of this study is then explored. Finally, the study limitations and future research prospects are discussed.

## Background

### The Influence of the COVID-19 Outbreak on College Students' Access to Information

The psychosocial cognition of college students' on COVID-19 is positively correlated with active coping while negatively correlated with negative coping. The college students with high cognition are likely to take active coping measures (13). This notion indicates that improving the cognition on the disease can aid individuals to respond positively. Approximately 84.0% of college students believe that the government has taken positive and effective measures, recognized the efforts made by the authorities and have confidence in the mandated epidemic prevention and control efforts (14). Purportedly, 95.8% of college students have collected information about COVID-19 *via* WeChat and microblog. Still, 83.0% of college students have collected information about COVID-19 epidemic situation *via* TV and radio. The emerging media, represented by WeChat and microblog, are characterized by instant communication, popularity of the main body and visualization of pictures and texts. Such platforms have broadened the learning channels of college students and meet the psycho-socio needs of college students. Thus, new media have become the main channel for college students to acquire epidemic information.

### The Influence of the COVID-19 Outbreak on College Students' Psychology

The psychological mood of college students is greatly affected due to rising pressures from diverse sources. For example, the implementation of strict home quarantine causes many college students to stay at home. Often characterized as in the psychological development stage, these students are likely to develop negative psychological emotions, such as depression, anxiety, and stress, due to undue pressures both from the epidemic and long hours of study. In serious cases, these emotions can even affect the students' health. Depending on the environmental circumstances, the prevalence rates of PTSD (Post-Traumatic Stress Disorder) and depression during the outbreak range from 2.7 to 9.0% (15). If college students are in their fourth year (the year of graduation), and live in seriously affected areas, then extreme fear is the key influencing risk factor causing psychological distress, followed by short sleep time.

Odriozola-Gonzz et al. (16) investigated the psychological wave of Spanish University of Valladolid during the outbreak. About 50.43% of the interviewees said that they were moderately affected, some even severely. Students majoring in the arts and humanities, social sciences and law had higher scores in anxiety, depression, and stress, whilst those majoring in engineering and architecture have lower scores. The clinical depression cases increased by 25–30 times due to the closure measures taken during the outbreak, and the number of Greek college students with suicidal thoughts increased by eight times (17). One third of people accepted the theory that COVID-19 was conspiracy. The study results especially emphasized the need to take specific intervention measures for the mental health of disadvantaged groups.

Put simply, investigating the knowledge of college students on COVID-19 and analyzing their attitudes toward epidemic prevention and control measures will be key to unlock and improve our understanding of how best to prevent and hinder the spread of COVID-19 among college students, as well as how best to plan on beneficial and impactful educational interventions for them (18). The COVID-19 had a greater impact on the psychology and sleep of college students (19, 20). Virtual mentorship pairing was the highest rated educational intervention suggested by first- and second-year medical students. The third- and fourth-year medical students had frequently cited virtual surgical skills workshops (21). The prevalence rates of anxiety and depression were 7.7 and 12.2%, respectively (22). The study results of (23) showed that the incidence of somatic symptoms amongst college students was 34.85 (mild, 26.26%; moderate, 8.59%). The incidence of somatic symptoms in primary school students was 2.39% (all mild).

Amongst the entire cohort, concern regarding COVID-19 was positively correlated with the occurrence of somatic symptoms. Somatic symptoms were likely amongst college students expressing greater concern regarding the threat to life and health posed by COVID-19 and the efficacy of prevention and control measures. Approximately 70% of learners reported that they were involved in e-learning during the lockdown. Most learners used android mobile in e-learning environments (24). Students have been facing various problems related to depression anxiety, poor internet connectivity and unfavorable study environment at home. Importantly, students who face enormous challenges in their studies during this pandemic come mainly from remote areas and marginalized sections of society.

The outbreak of the epidemic increased the loneliness of overseas students and affected their career development (25). With the outbreak of the epidemic, a stable family income and living with parents could effectively reduce anxiety (26). Economic effect aside, the impact on daily life and academic delay are also positively correlated with anxiety, whilst social support is negatively correlated with anxiety (27). Savitsky et al. (28) studied the influence of COVID-19 on the psychology of nursing students. Their results showed that female gender, lack of personal protective equipment (PPE) at work and being a parent were significantly associated with high anxiety scores. Zhang et al. (29) studied the influence of COVID-19 on the psychology of international students in Medical Bachelor, Bachelor of Surgery of Zhejiang University. Even though students' knowledge on Traditional Chinese Medicine (TCM) and their discussion and consultation behaviors have been significantly improved, and students' understanding on the necessity of TCM has been significantly enhanced based on the online Chinese medicine courses, most students still preferred face-to-face classroom learning compared to online learning.

### Analyzing Causes of Major IPO

In general, researchers believe that major IPO is the product of the comprehensive action of various social factors, and they can explore the causes from various aspects, including the subject and object of public opinion, media, social environment, contingency factors and the manifestation of public opinion (30).

Briefly, some scholars analyzed the causes of IPO from four dimensions, including political and economic reasons, social environment and hot events, false and bad information dissemination, the strong media influence of new media era (31, 32). Also, some scholars analyzed the configuration of the influencing factors on IPO and found that maintaining high sensitivity of information source, high participation of Internet users and/or high media activity and high government intervention can stimulate the generation of a heat index of high public opinion (33, 34). In the study reported herein, we investigated the causes of generating a major IPO from specific fields.

The first cause includes objective and realistic factors. The IPO is an inevitable product of the frequent occurrence of social public events. The sudden public events will cause great psychological influence on the public (35, 36). The second comprises government intervention factors. If the timeliness of government intervention is worse, and the intervention level is high, then the heat of IPO is high. The heat of public opinion events responded by the government *via* press conference and social media is high (37, 38). The third involves the psychological factors of public opinion subjects. The optimistic mood of netizen and the pessimism of the government can bring favorable results to the development of public opinion, and the influence of the emotional state and intensity of the government is great (39).

## Research Model and Hypotheses

A key characteristic of modern democratic society is social participation, which refers mainly to the direct participation and influence of the public on social and public affairs (40). The social participation mechanism, a type of participatory democracy, is a process and approach to achieve societal growth *via* active participation into social development activities based on the public's concern for their own interests and their conscious recognition on social public interests and public objects (41). Hence, the following hypotheses are put forward in this study:

### H1: AA Has a Significant Positive Influence on the Consciousness of One's Social Participation

Adult attachment (AA) is a sustained and long-term emotional connection between adult individual and current peers and a stable interpersonal interaction style of the individual (42). Hence, it can influentially affect the consciousness of one's social participation in a positive manner.

### H2: SM Has a Significant Positive Influence on the Consciousness of One's Social Participation

Different scholars have conceptualized how social motivation (SM) can influence or affect people differently. While Hilvert-Bruce et al. (43) believed that SM is the basic force driving people's social behavior, and Hernandez et al. (44) believed that SM is an internal motivation that promotes individual activities to achieve a certain goal. Based on the dictionary of psychology (45), we purport that SM is the psychological tendency of individuals to engage in certain activities to meet their own social needs, which is directly related to their social needs, but not to their physiological needs. Hence, it can surely influence the consciousness of one's social participation.

### H3: IAD Has a Significant Positive Influence on the Consciousness of One's Social Participation

Information affective disposition (IAD) refers to the college students' inner emotional attitude toward the text when they are reading the major epidemic information. Therefore, it too can certainly influence the consciousness of one's social participation as well as one's emotion, which is linked invariably to H4 below.

### H4: IAD Has a Significantly Positive Influence on Emotional Motivation (EM)

Emotional motivation (EM) refers to the condition wherein people release public crisis information to express emotions. After the outbreak of a major epidemic, the public's psychology, emotion, and belief will be affected to different extents. Individuals in such crisis situations are prone to have panic, depression, anxiety, and other negative emotions. The public can reduce anxiety and vent discontent based on the information transmission *via* the Internet (46, 47). The public will often express their feelings to relatives, friends, and even strangers *via* real-time dissemination of information to seek comfort, understanding and spiritual support to alleviate the inner uneasiness in the crisis situation or release the dissatisfaction with the crisis management stakeholders (48). Hence, as noted in the discussion for H3, IAD will certainly also influence one's emotional motivation.

### H5: AA Has a Significantly Positive Influence on EM

Emotional catharsis is a form of public right of discourse and participation, which is conducive to the self-regulation of personal emotions in crisis situations, seeking self-comfort and psychological balance (49). Not surprisingly, we can speculate that AA, which projects one's attachment to others' emotions and related feelings, to influence one's EM significantly.

### H6: Risk Pereption Has a Significantly Positive Influence on EM

Risk relates to the consideration of the possible loss or injury caused by objective facts (50, 51). EM has a significant influence on individual participation in social activities (52). Put together, risk perception, especially that is related to the risk that the dissemination of error messages will possibly lead to crime, will generally be heighten for the public when the loss or injury caused may be serious. Accordingly, such risk perception will assert an influence also on one's EM.

### H7: SM Has a Significantly Positive Influence on the Dissemination Willingness

As indicated, SM or one's psychological tendency to engage in certain activities to meet one's social needs can play a critical role in the dissemination of information. Since the 1970s, emotion study has blossomed into a new discipline. Exploring key influencing factors on microblog users' participation in interaction, Stuart and Martin (53) found that perceived usefulness and expectation confirmation significantly affect users' willingness to use.

### H8: The Consciousness of Social Participation Has a Significantly Positive Influence on the Dissemination Willingness

Weingart et al. (54) analyzed the intention of dissemination vis-à-vis the theory of SM and pointed out that personal needs, interest promotion, self-expression and self-actualization are the main motivations to influence the intention of dissemination.

### H9: EM Has a Significant Positive Influence on the Dissemination Willingness

As well, the theory of EM proposed by Auguste Comte, a famous French philosopher and the founder of sociology and positivism, tells us that emotion, behavior, and talent comprise humanity. Emotion is a decisive factor; as such, human emotion drives human reason and activity goal. Put simply, human behavior is mainly driven by EM. Hence, it is expected that EM will critically influence one's willingness to disseminate information.

### H10: Risk Perception Has a Significant Positive Influence on the Dissemination Willingness

Cao et al. (55) analyzed the influencing factors of users' willingness to disseminate enterprise microblog information from the perspective of enterprise microblog marketing. Their results showed that the benefits and reputation increase positively affected their intention of dissemination.

### H11: EM Plays a Mediating Role in the Relationship Between Emotional Orientation and Dissemination Willingness

Some scholars also analyzed users' motivation from the perspective of a microblog generation content (56). The research conclusions from Hsu and Lin (57) pointed out that the entertainment role, altruism and promotion of honor had a positive influence on the generation behavior of bloggers. The promotion of status positions and the sense of belonging had a significant influence on the continuous generation and sharing of a user content. Park et al. (58) indicated that the interest, usefulness, altruism, and identity have a significant influence on the generation behavior of video users.

### H12: Social Participation Consciousness Plays a Mediating Role in the Relationship Between SM and Dissemination Willingness

The social participation mechanism, a participatory democratic process and approach, achieves the growth and development of society *via* active participation into social development activities moderated by the concern of the public over their own interests and awareness respecting social public interests and public affairs (59). This approach is a code of conduct and norm for the entire society to discuss, consult and make decisions on social affairs (60, 61).

### H13: SM Plays a Moderating Role in the Relationship Between Risk Perception and Dissemination Willingness

With the frequent occurrence of crisis and the development of democracy, the consciousness of crisis, political consciousness and the sense of participation are gradually strengthening. In public emergencies, public participation has become a new norm (62). The motivation of crisis information dissemination in a mobile Internet environment includes egoistic (emotional and social) motivation and altruistic (informational) motivation (63).

## Research Methodology

### Sampling and Data Collection

In this study, the questionnaire survey method was used to conduct empirical research and data collection. Chinese college students served as the objects investigated.

The questionnaire, designed according to the unified standards and requirements, was distributed online *via* WWW.WJX.CN, an online research data collection platform located in Changsha, China. This platform executes the questionnaire administration process and provides powerful data collection, storage, and analytical tools to deeply explore the value of data being collected. Presently, reported statistics on WWW.JX.CN indicated that about 83.15 million users have responded to over 6.55 billion questionnaires.

In this study, a total of 438 questionnaires were distributed, and 319 valid questionnaires were collected with an effective respond rate of 72.83%. Descriptive statistics indicated that, amongst the valid samples, 48.6% were male, and 51.4% were female.

According to data shown in Table 1, the distribution of the objects investigated was as follows:

**Table 1:** Demographic characteristics of valid samples.

**Variables**	**Categories**	**Frequency**	**Percentage**
Gender	Male	155	48.6%
	Female	164	51.4%
Grade	Freshmen	92	28.8%
	Sophomore	113	35.4%
	Junior	79	24.8%
	Senior	35	11%
Specialized subject	Liberal arts	117	36.7%
	Science	111	34.8%
	Engineering	84	26.3%
	Arts and sports	7	2.2%
Online time every day	1–2 h	13	4.1%
	3–4 h	91	28.5%
	5–6 h	121	37.9%
	More than 6 h	94	29.5%
Frequency of paying attention to public opinion	Every day	91	28.5%
	Often	174	54.5%
	Occasionally	52	16.3%
	Less	2	0.6%

With respect to the “Grade” variable, first-year students (freshmen) accounted for 28.8%, second-year students (sophomore) for 35.4%, third-year students (junior) for 24.8% and forth-year students (senior) for 11%.

In term of specialized subject, liberal arts students accounted for 36.7%, those with science major for 34.8%, those with engineering major for 26.3%, and students of arts and sports for 2.2%.

As for time spent online: 4.1% of college students surfed the Internet for 1–2 h daily, 28.5% surfed the Internet for 3–4 h daily, 37.9% surfed for 5–6 h daily, and 29.5% of the students surfed for more than 6 h daily.

Regarding the frequency of paying attention to COVID-19 public opinion: 28.5% of college students paid attention to it daily, 54.5% of college students often paid attention to it, 16.3% of the students paid attention to it occasionally, and 0.6% of them paid less attention to it.

### Questionnaire and Measurements

The questionnaire adapts multiple items from previously well-developed scales. It was also designed in strict accordance with the translation–back translation procedure. Altogether, the questionnaire has been appropriately adjusted through pretesting relevant questions, recognizing the actual situation of Chinese college students.

The comprehensibility of problem description was ascertained *via* interviews. The pre-survey questionnaire was issued according to the results. The questionnaire was further modified vis-à-vis the feedback of the pre-survey. The formal questionnaire was finally formed. Besides the investigation on the basic information of college students, the Likert 7-scale was used to record the attitude of objects investigated toward various questions provided in the questionnaire. “1” represents strongly disagreed; “7” indicates strongly agreed.

The Affective Disposition (AD) Scale was adopted from the (64) questionnaire with the scale comprising two question items. The average score of all items in the scale was regarded as the AD measurement score. The AD was significant when the score was high. The Dissemination Willingness Scale was adopted from the (52) questionnaire with its scale having three question items. The average score of all items in the scale was regarded as the measurement score. The dissemination willingness of the objects investigated was strong when the score was high. The Emotional Motivation (EM) Scale was derived from the (65) questionnaire. This scale contained two question items. The average score of all items in the scale was regarded as the EM measurement score. The EM of individuals was strong when the score was high.

The Adult Attachment (AA) Scale was derived from the (66) questionnaire; accordingly, this scale has three question items. The average score of all items in the scale was regarded as the AA measurement score. The AA of individuals was strong when the score was high. The Social Motivation (SM) Scale was derived from the (44) questionnaire, which contained two question items. The average score of all items in the scale was regarded as the SM measurement score. The SM of individuals was strong when the score was high. The Social Participation Consciousness Scale was drawn from the questionnaire designed by (67). This scale contained three question items. The average score of all items in the scale was regarded as the Social Participation Consciousness measurement score. This characteristic of individuals was strong when the score was high. In the Risk Perception Scale, the questionnaire designed by (68) was adopted. This scale contained two question items. The average score of all items in the scale was regarded as the risk perception measurement score. The risk perception of individuals was strong when the score was high. The final measurement items of seven constructs may be found in the Appendix.

### Reliability and Validity Analysis

The reliability and validity of risk perception, dissemination willingness, AA, EM, and SM were analyzed by SPSS24.0 with results shown in Table 2. This table illustrates that the Composite Reliability (CR) of all potential variables to be more than 0.8, and Cronbach's α coefficients of all variables are all higher than the recognized minimum level (0.6). This indicates that all the scales have good reliability.

**Table 2:** Reliability analysis.

**Construct**	**Item**	**Factor loading**	**Cronbach's α**	**CR**	**AVE**
Affective disposition of information (ADI)	ADI 1	0.821	0.677	0.858	0.752
	ADI 2	0.911			
Dissemination willingness (DW)	DW 1	0.852	0.776	0.870	0.690
	DW 2	0.855			
	DW 3	0.783			
Emotional motivation (EM)	EM 1	0.788	0.656	0.847	0.736
	EM 2	0.922			
Adult attachment (AA)	AA 1	0.852	0.792	0.877	0.703
	AA 2	0.820			
	AA 3	0.844			
Social motivation (SM)	SM 1	0.844	0.622	0.841	0.726
	SM 2	0.860			
Consciousness of social participation (CSP)	CSP 1	0.855	0.734	0.849	0.654
	CSP 2	0.839			
	CSP 3	0.726			
Risk perception (RP)	RP 1	0.878	0.707	0.872	0.773
	RP 2	0.881			

Exploratory factor analysis was used to test the construct validity of the scale. The factor load of each item corresponding to all variables was greater than the threshold value of 0.7, thereby indicating a good construct validity of the scale (69).

The average variance extracted (AVE) of each variable is more than 0.65, thereby indicating that the convergence validity of the table is good (70). Table 3 illustrates that the square root of AVE of each variable to be more than the correlation coefficient of this variable vis-à-vis other variables. A good discriminant validity exists amongst the variables (71). In summary, the scales used in this study have good validity.

**Table 3:** Validity analysis.

	**DW**	**ADI**	**EM**	**AA**	**SM**	**CSP**	**RP**
Dissemination willingness	0.831						
Affective disposition of information	0.121	0.867					
Emotional motivation	0.199	0.418	0.858				
Adult attachment	0.036	0.360	0.405	0.839			
Social motivation	0.309	0.019	0.078	−0.005	0.852		
Consciousness of social participation	0.295	−0.004	−0.006	−0.142	0.322	0.809	
Risk perception	0.086	0.178	0.335	0.284	0.058	−0.009	0.879

## Data Analysis and Results

The Partial Least Squares (PLS) method has been deployed for analyzing the study data. PLS is a new multivariate data analytical method. Compared to other methods, PLS is more reliable and stable in the calculation, making it suitable for the analysis on small sample data. Importantly, PLS can achieve simultaneous modeling and prediction and realize the comprehensive simplification of the multivariable system and the correlation analysis between the two groups of variables, which can effectively solve the collinearity problem.

The main purpose of PLS is to construct the regression models of multiple dependent and independent variables. PLS can flexibly set the types of external relations in the structural equation whilst constructing the model according to the actual situation, that is, supporting constitutive and reflective models. Here, the research model is analyzed *via* the Smart PLS3.0 software.

### Structural Model Test

**Figure 1:**
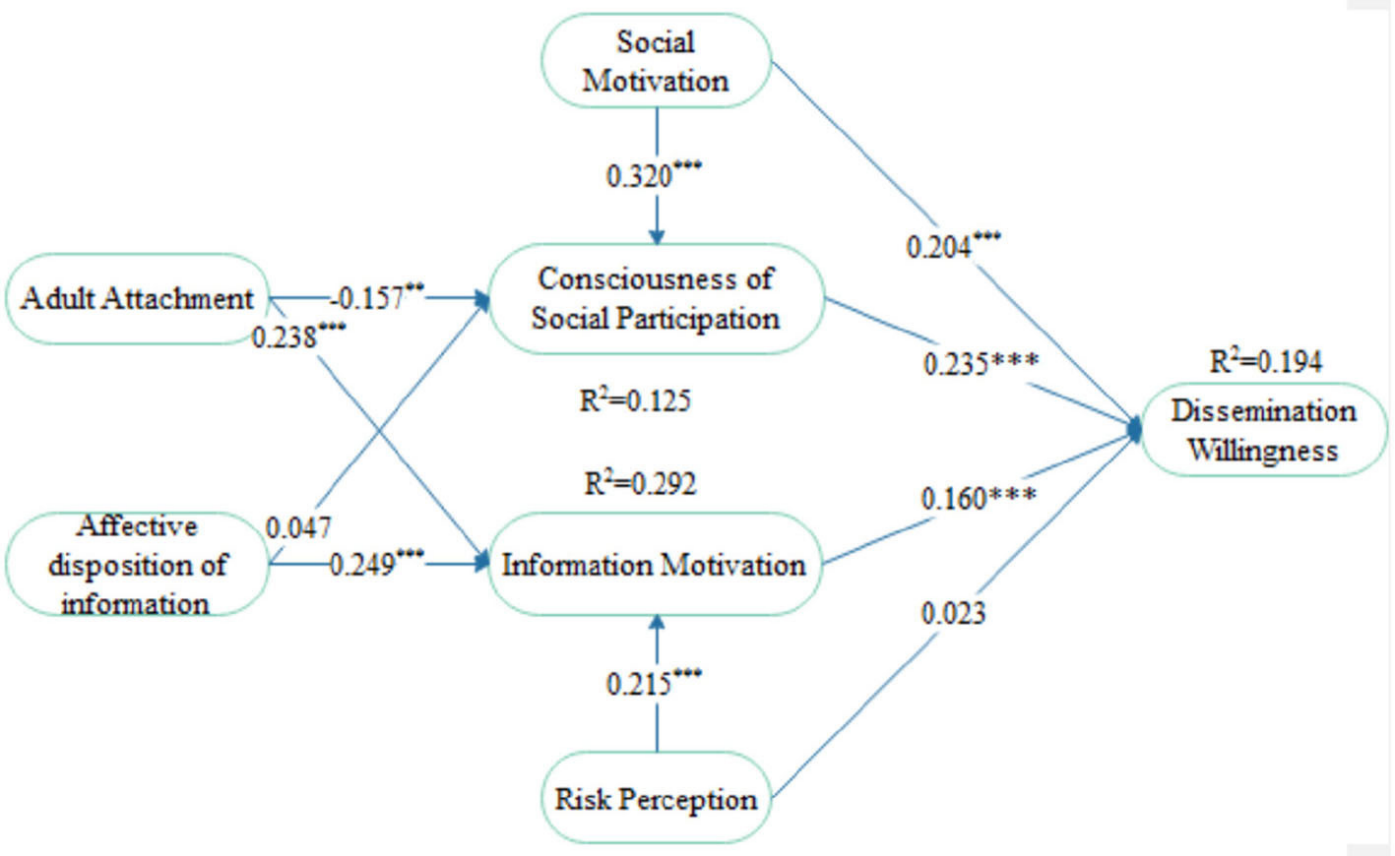
Model path and significance level. **p < 0.05, ***p < 0.01.

Path coefficient represents the strength of the relationship between independent and dependent variable. The analytical results of the path coefficient for the research model being validated are shown in Figure 1 and Table 4.

**Table 4:** Hypothesis test results.

**Hypothesis**	**Path**	**Means**	**SD**	***t*-Value**	***P***
H1	AA → CSP	−0.160	0.063	2.483	0.013
H2	SM → CSP	0.324	0.051	6.323	0.000
H3	ADI → CSP	0.048	0.060	0.774	0.439
H4	ADI → EM	0.293	0.057	5.114	0.000
H5	AA → EM	0.241	0.057	4.182	0.000
H6	RP → EM	0.218	0.058	3.678	0.000
H7	SM → DW	0.205	0.056	3.636	0.000
H8	CSP → DW	0.240	0.060	3.890	0.000
H9	EM → DW	0.159	0.054	2.967	0.003
H10	RP → DW	0.024	0.055	0.409	0.683

From the analysis results, among the first ten (10) primary hypotheses advanced on the structural model test, eight (8) were supported. R^2^ represents the variance variability of the dependent variable explained by the independent variable. In this study, bootstrapping repeated sampling method is used to calculate the *t*-value of the significance test based on the selection of 2,000 samples. The degree of interpretation of consciousness of social participation, EM and dissemination willingness are 0.426, 0.427, and 0.545 respectively, thereby indicating that the model has a good interpretation effect (71).

**Figure 2:**
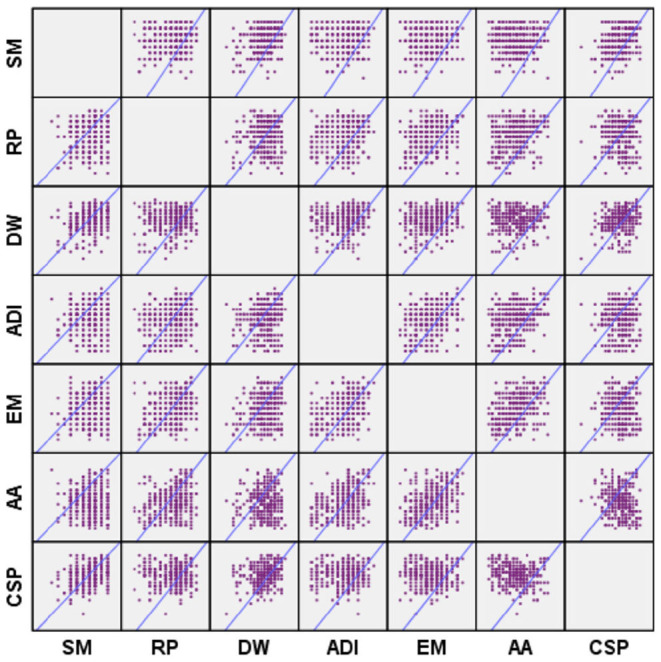
Distribution plot of hypothesis testing.

As quantified in Table 4, bootstrapping method has been used to test the significance of the path coefficient of the structural model. As depicted in the distribution plot of hypothesis testing shown in Figure 2, there is a significant correlation between the seven variables in the hypothesis test.

First, AA (adult attachment) has a significantly positive influence on the consciousness of social participation (β = 0.157, *t* = 2.438), affirming *Hypothesis H1*. SM (social motivation) also has shown a significantly positive influence on the consciousness of social participation (β = 0.320, *t* = 6.323), confirming *Hypothesis H2*. Conversely, IAD (information affective disposition) has no significant positive effect on the consciousness of social participation (β = 0.047, *t* = 0.774), inferring that *Hypothesis H3* is not supported.

Even so, for hypotheses *H4 through H6*, AD has shown to have a significant positive influence on EM (emotional motivation) (β = 0.249, *t* = 5.114); hence, *Hypothesis H4* is supported. AA also has a significant positive influence on EM (β = 0.238, *t* = 4.182), confirming *Hypothesis H5*. As well, risk perception has a significant positive influence on EM (β = 0.215, *t* = 3.678), thereby confirming *Hypothesis H6*.

In term of hypotheses *H7 through H10*, results of the data analysis showed that SM also has a significant positive influence on the dissemination willingness (β = 0.204, *t* = 3.636). Therefore, *Hypothesis H7* is supported. As well, the consciousness of social participation has a significant positive influence on dissemination willingness (β = 0.235, *t* = 3.890). Hence, *Hypothesis H8* is also supported. Additionally, EM has a significant positive influence on dissemination willingness (β = 0.160, *t* = 2.967), thereby confirming *Hypothesis H9*. However, risk perception has no significant influence on dissemination willingness (β = 0.023, *t* = 0.409). As such, *Hypothesis H10* is not supported.

### Mediating Effect Test

In this study, we conducted a mediating effect hypothesis test by using a three-step mediating regression analysis as proposed by (72). In the first step, AD is used to regress dissemination willingness; next, AD is used to regress EM. Finally, in the third step, AD and EM were used together to regress the dependent variable (dissemination willingness). The regression coefficient must be significant in the first and second steps. In the third step, if the regression coefficient of EM is significant, and the regression coefficient of the independent variable AD is insignificant, then EM plays a complete mediating role. If the regression coefficients of AD and EM are significant, but the regression coefficient of AD becomes weak, then EM plays a partial mediating role.

This study analyses the mediating role of EM in the relationship between AD and dissemination willingness *via* the bootstrapping method on the basis of the suggestions of (73). Here, 5,000 times of repeated sampling is adopted in the bootstrap analysis to construct a 95% confidence interval for bias correction.

Table 5 demonstrates that the indirect effect of EM in the relationship between AD and dissemination willingness is 0.051 (CI = [0.014,0.088]), and its bootstrap 95% confidence interval does not include zero. This result indicates that the mediating effect of EM between AD and dissemination willingness is significant. Therefore, *hypothesis H11* is supported. The indirect effect of the consciousness of social participation in the relationship between SM and dissemination willingness is 0.070 (CI = [0.038, 0.127]), and its bootstrap 95% confidence interval does not include zero. This result indicates also that the mediating effect of the consciousness of social participation in the relationship between social motivation and dissemination willingness is significant. *Hypothesis H12* is confirmed.

**Table 5:** Mediating effect of emotional motivation and consciousness of social participation.

**Indirect effect**	**Estimated value**	**97.5% CI**		**Conclusion**
		**Lower**	**Upper**	
Affective disposition of information → emotional motivation → dissemination willingness	0.051	0.014	0.088	Mediation effect
Social motivation → consciousness of social participation → dissemination willingness	0.070	0.038	0.127	Mediation effect

### Moderating Effect Test

First, the centralization treatment is conducted for the independent and moderating variables to test the regulatory effect of SM on the relationship between risk perception and dissemination willingness. Then, the product terms of SM, risk perception and dissemination willingness are constructed to conduct a multilevel regression analysis. SM has a significant moderating effect on the relationship between risk perception and dissemination willingness.

**Figure 3:**
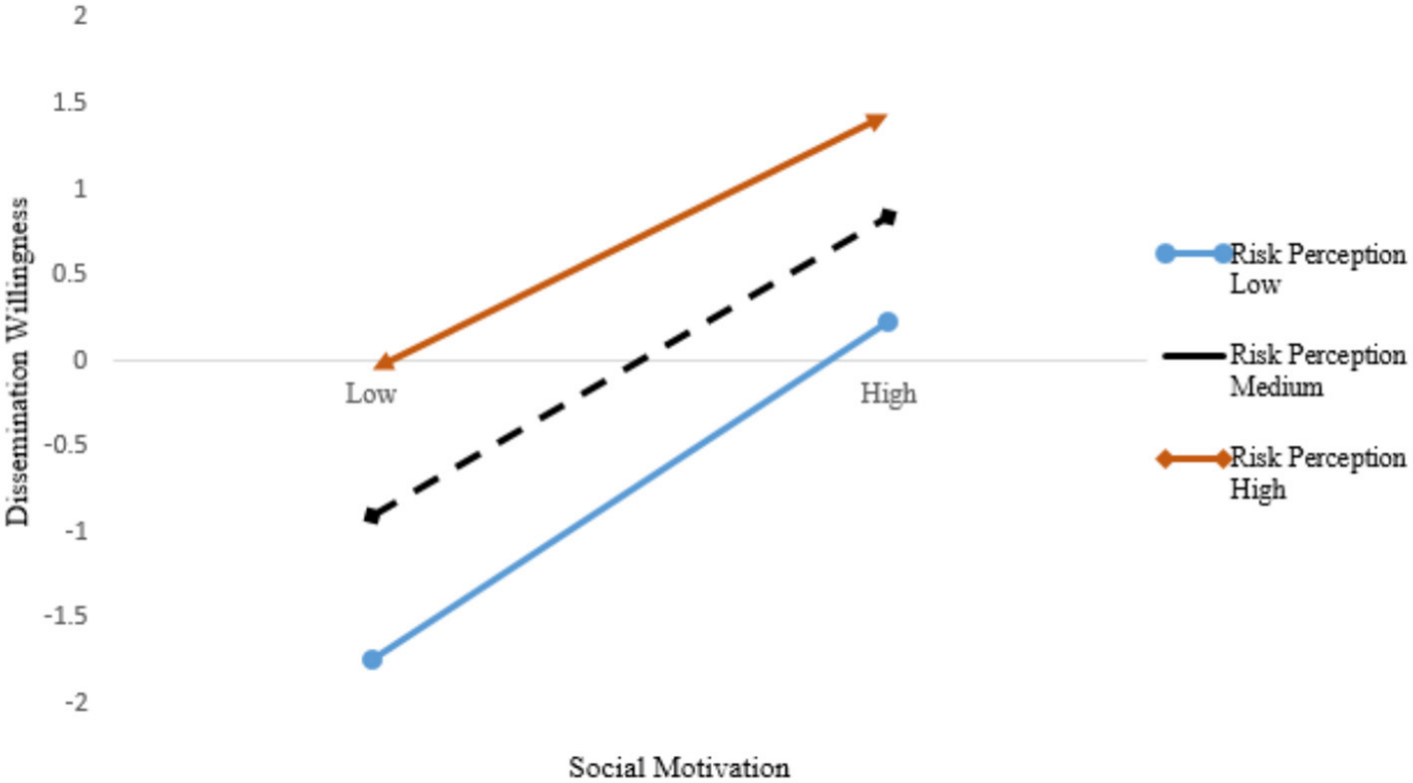
Moderating effect of risk perception on the relationship between social motivation and dissemination willingness.

In the study, risk perception is divided into high, medium, and low situations to clearly show the role of the moderating variables. Excel is used to draw the influence of SM on dissemination willingness in high, medium, and low risk perception. The main effect is 0.87; the moderating variable effect is 0.724; and the moderating effect is −0.121. The moderating effects are shown in Figure 3. Our results show that the influence of SM on dissemination willingness is reduced when the risk perception is high, confirming *Hypothesis H13*.

## Discussion

AA and SM have significant positive effects on the consciousness of social participation, *Hypothesis H1and H2* are demonstrated. Nonetheless, the positive influence of AD on the consciousness of social participation is not confirmed as *Hypothesis H3* is not supported. This situation is likely (and primarily) due to college students being in the period of psycho-physio development with the lack of rational judgment on the development of social affairs. Hence, their ability to judge the reliability and certainty of information to effectively strengthen the education of epidemic-related knowledge should be reinforced to make them understand better the epidemic situation based on a rational and scientific approach. Adopting such an evidence-based strategy, college students will then be aided to adopt positive coping styles and maintain physical and mental health.

SM, the consciousness of social participation and EM have significant positive effects on dissemination willingness. *Hypothesis H7, H8, H9* have been demonstrated. Nonetheless, the positive influence of risk perception on dissemination willingness is not confirmed as *Hypothesis 10 is not affirmed*. Additionally, this analysis indicates the varying characteristics of college students. The self-health level of people and the psychological processing mode of external emergencies are uniquely different (74). This situation will lead to varying risk perception on crisis events, thereby resulting in different coping methods. College students should have a theory-based understanding on their own physical health level and objective knowledge of their own psychological processing mode. These students should formulate a specific epidemic response mode based on their specific actual situations to alleviate the worries and anxiety caused by the epidemic.

AD, AA, and risk perception have significant positive effects on EM. *Hypothesis H4, H5, H6* have been demonstrated. Public opinion is generated around intermediary social events (75). Hence, college students' preference for events can directly affect their behaviors of expressing their opinions, ideas, or emotions. However, affective disposition and risk perception are always the decisive factors to attract college students' attention.

EM plays a mediating role in the relationship between AD and dissemination willingness. *Hypothesis H11* has been demonstrated. College students will be worried and panic about the spread of the epidemic (76), and they will also show a concerning attitude toward the government's measures to prevent and control the epidemic, which will affect dissemination willingness. The consciousness of social participation plays a mediating role in the relationship between SM and dissemination willingness. *Hypothesis H12* has been demonstrated. This result is obtained as college students have a strong sense of participation in social activities. Students hope to help their friends and relatives understand the extent and severity of the epidemic spread *via* the dissemination of public opinion information. The consciousness of social participation has a mediating effect on dissemination willingness. SM moderates the relationship between risk perception and dissemination willingness. *Hypothesis H13* has been demonstrated.

COVID-19 is characterized by rapid spread, high infection, and wide spreading scope. College students have a deep understanding on the risk of epidemic development and hope to help their friends, classmates, and family members through their own efforts. SM has a great effect on the relationship between risk perception and dissemination willingness.

## Implications

In this study, we have constructed a research explanatory model on the spread of major epidemic IPO and proposed the coping strategies. These initiatives can provide reference and insight into the current and future IPO governance practice in major epidemic situation.

More importantly, these initiatives provide theory-based thinking for further enriching and deepening the research on IPO governance with major epidemic as the typical sudden public health practice.

### Theoretical Implications

This study unveils key influencing factors on IPO dissemination in major epidemic prevention and control from two primary dimensions, namely, SM and EM. The conclusion of this study compensates for the current studies on group factors and the mechanism of social participation consciousness. This study enriches IPO research with several theoretical insights, expanding the research scope of students' information behavior theory in the process of spreading public opinion on the epidemic.

Studies have theoretically verified that EM plays a mediating role in the relationship between AD and dissemination willingness; consciousness of social participation plays a mediating role in the relationship between SM and dissemination willingness; and SM moderates the relationship between risk perception and dissemination willingness (77). The research model offers insight and analysis on the governance mechanism of IPO in the prevention and control of major epidemic to supplement the understanding of current studies on the role of governance mechanism.

### Managerial Implications

The governance effect of IPO determines the guidance of public opinion in the context of sudden events, which is directly related to social stability. Effective measures for IPO governance must be determined.

The outcome of this effort is of great relevance to the governance of IPO in the prevention and control of major epidemic and of applicable significance to advancing the government's information governance ability. We should strengthen the guidance of public opinion in major epidemic prevention and control and fully build and make good use of all types of information transmission paths. The spread of IPO is a group activity, rather than an individual action. This situation has a strong external effect.

In the context of public emergencies, IPO has an inciting force to the group. When dealing with the IPO in major epidemic prevention and control, it also tests the ability of prevention and response to group events. We must set up a network warning in advance, implement information communication channels in time and ensure the real-time interaction of information. These steps can reduce the possibility of Internet users to believe and spread rumors to a certain extent, thereby promoting the public to form self-awareness and moral obligations and maintain a harmonious network environment.

## Limitations and Future Research

In this study, several key limitations may be observed. First, the study is based on the risk of spreading COVID-19 and users' risk perception. Users' habits and preference of public opinion dissemination, the characteristics of subjects and objects of spreading public opinion and other influencing factors have not been included in this study.

Importantly, the model established here is just a preliminary prototype, and its prediction accuracy may still need enhancement. After further improvement of the measurement scheme and simulation means, the relevance and applicability of the studied model will be greatly improved. As well, given that the objects investigated are college students, restrictions on age and educational level may exist. In the future, we anticipate broadening the selection of the research objects and comprehensively consider the differences amongst groups of different regions, occupations, and ages.

Finally, public opinion dissemination in an era of major epidemic prevention and control will also be intervened by related government and health departments; for example, the spread of proper health knowledge, criticism, and education to fight malicious rumor makers *via* appropriate social media channels by relevant authorities may improve the dissemination of public opinion. In addition to the sources, objects and contents of dissemination, the third-party intervention can and should be further studied in the future.

## Data Availability Statement

The raw data supporting the conclusions of this article will be made available by the authors, without undue reservation.

## Ethics Statement

The studies involving human participants were reviewed and approved by Wuhan Business University Ethics Committee. The patients/participants provided their written informed consent to participate in this study.

## Author Contributions

Overall supervision, advisor, and final approval of submitted work by LL. Primary research data and statistical analytic work by PY. Review of research and final submission by JT. All authors contributed to the article and approved the submitted version.

## Funding

This work was supported by the Hubei Social Science Research Fund [19ZD074].

## Conflict of Interest

The authors declare that the research was conducted in the absence of any commercial or financial relationships that could be construed as a potential conflict of interest.

## Publisher's Note

All claims expressed in this article are solely those of the authors and do not necessarily represent those of their affiliated organizations, or those of the publisher, the editors and the reviewers. Any product that may be evaluated in this article, or claim that may be made by its manufacturer, is not guaranteed or endorsed by the publisher.

## Appendix: Constructs and Items

**1. Affective Disposition (AD) of Information**

The information on the epidemic situation clearly expresses the questioning attitude to the government's prevention and control measures.

The information on the epidemic situation reveals dissatisfaction with relevant departments.

**2. Dissemination Willingness (DW)**

If my friend is interested, then I am willing to spread the public opinion of campus emergencies to him/her.

If I find a discussion area similar to the public opinion of campus emergencies, then I will forward the information.

I will forward the public opinion of campus emergencies to my families and friends.

**3. Emotional Motivation (EM)**

I would like to express my concern and panic about the spread of the epidemic.

I would like to express my doubts about the relevant government departments and their measures.

**4. Adult Attachment (AD)**

I find that when I need help, no one would help me.

I want to be close to others, but I am afraid that I will be hurt.

Being close to others makes me feel a little uncomfortable.

**5. Social Motivation (SM)**

I want to express my concern for the epidemic.

I want to express my concern for the people affected by the epidemic.

**6. Consciousness of Social Participation (CSP)**

I often take part in some social activitiess.

I believe that social activities will enrich my life.

I will actively share my experience in social activities.

**7. Risk Perception (RP)**

I think that the prevalence and spread of this epidemic are difficult to control.

I think that this epidemic disease is difficult to treat.

## Glossary

1. Adult Attachment (AA): is a sustained and long-term emotional connection between adult individual and current peers and a stable interpersonal interaction style of the individual. Adult attachment behavior is developed and maintained through a cognitive system that creates internal working models of relationships with others through relational experience (Bowlby's attachment theory, 1982/1969).
2. Affective Dispositions (AD): are dynamic, changeable, teachable, cultivable, and developmental qualities which are socially constructed and individually shaped (NCATE, Professional standards for the accreditation of teacher preparation institutions, Program 2008; Obara, Processes of Disposition Development in K-5 Teachers, 2009). AD could be categorized as desirable and undesirable.
3. Social Motivation (SM): is the psychological tendency of individuals to engage in certain activities to meet their own social needs, which is directly related to their social needs, but not to their physiological needs.
4. Emotional Motivation (EM): refers to the condition wherein people release public crisis information to express emotions. After the outbreak of a major epidemic, the public's psychology, emotion, and belief will be affected to different extents.
5. Average Variance Extracted (AVE): measure the average variance shared between a construct and its measures, and by calculating the correlations between different constructs. A matrix can then be constructed where the square root of AVE is in the diagonal, and the correlations between the constructs are in the off diagonal. For adequate discriminant validity, the diagonal elements should be greater than the off-diagonal elements in the corresponding rows and columns (70).
